# Effect of Moisture on Mechanical Properties and Thermal Stability of Meta-Aramid Fiber Used in Insulating Paper

**DOI:** 10.3390/polym9100537

**Published:** 2017-10-22

**Authors:** Fei Yin, Chao Tang, Xu Li, Xiaobo Wang

**Affiliations:** 1 College of Engineering and Technology, Southwest University, Chongqing 400715, China; yf1992@email.swu.edu.cn (F.Y.); lixuqq@email.swu.edu.cn (X.L.); xiaobo@email.swu.edu.cn (X.W.); 2 School of Electronics and Computer Science, University of Southampton, SO171BJ Southampton, UK

**Keywords:** meta-aramid fibers, moisture, hydrogen bond, mean square displacement, free volume

## Abstract

Seven composite models of meta-aramid fibers with different moisture contents were studied using molecular dynamics simulation. The effects of moisture on the thermal stability and mechanical properties of the fibers and their mechanisms were analyzed, considering characteristics such as hydrogen bonding, free volume, mean square displacement, and mechanical parameters. The simulation results showed that the large number of hydrogen bonds between water molecules and meta-aramid fibers destroyed the original hydrogen-bond network. Hydrogen bonds between the molecular chains of meta-aramid fibers were first destroyed, and their number decreased with increasing moisture content. The free volume of the fibers thereby increased, the interactions between fiber chains weakened with increasing moisture content, and the fiber chain movement intensified accordingly. The ratio of diffusion coefficients of the water molecules to moisture contents of the composite models increased linearly, and the water molecule diffusion increased, which accelerated the rate of damage to the original hydrogen-bond network of the meta-aramid fibers and further reduced their thermal stability. In general, the mechanical properties of the composites were negatively related to their moisture content.

## 1. Introduction

Oil-paper systems comprise the main insulation structures of oil-immersed power transformers. In long-term operation of a transformer, the insulating oil will decompose to produce water. Moisture accelerates aging of the insulating paper and decreases its mechanical, thermal stability, and dielectric properties [1,2]. During long-term operation of oil-paper transformer insulation systems, the moisture content of the paper will gradually increase to a certain value and eventually reach equilibrium with the moisture in the transformer oil. One of the main degradation products of paper is cellulose. Moisture increases the probability of fracture of the cellulose molecular chains under combined action with other degradation products [3,4,5]. Simultaneously, a large number of water molecules can form space bubbles at high temperature, which further deteriorate the properties of the insulating paper. The effect of moisture is also reflected in the reduction of the degree of polymerization of these large polymers, in the acceleration of the aging process of the insulating paper, and in the quantity of hydrogen ions in the paper. A hydrolytic catalyst is produced by these ions, which will accelerate cellulose hydrolysis. In this respect, studies have shown that a doubling of the moisture content causes a 50% decrease in the mechanical properties of insulating paper [6,7]. Numerous studies that have been conducted on the mechanical and electrical properties of transformer insulation systems indicate that moisture is one of the most important factors affecting transformer performance [8,9,10].

Aromatic polyamide macromolecules are high-performance materials that have been widely used in oil-paper insulating systems. A large amount of research by Chinese scholars on the thermal degradation of aramid fibers has shown that these fibers have high heat resistance, a service life exceeding 10 years at 180 °C, and good mechanical properties that are maintained at temperatures up to 300 °C [11,12]. Although aramid fibers have excellent performance, their mechanical properties deteriorate under conditions of long-term use that may imply thermal aging, surface damage, and cracking. Research of Jain, A. et al. [13] on thermal aging of Nomex fiber showed that temperature and time of exposure to air were the main factors influencing fiber's thermal degradation. Villar-Rodil, S. et al. studied the pyrolysis behavior of Nomex aramid fiber and the properties of the thermal degradation product. They showed that quality loss due to thermal aging of the fiber was divided into three stages [14]. 

Improvements in industrial technology have led to improved chromaticity, light resistance, and fatigue resistance of meta-aramid fibers. Numerous studies show that the properties of high molecular mass polymers [15,16,17,18,19] can be effectively improved by adopting technologies such as nanometer modification and grafting. Zhao, L.H. et al. [20] combined the aramid fiber with other materials. Chen, L. et al. [21] modified the aramid fiber by low-temperature solution copolymerization, which improved the service life, heat resistance, and mechanical properties of the resulting composite paper. Bai, G. et al. [22] effectively lowered the conductive current of the aramid fiber and improved the alternating-current (AC) breakdown field by modifying 1313 fibers with nanometer silicon nitride. By using grafting, Wang, H.H. et al. [23] increased the stretching, bending, and impact strengths of a composite material of the aramid fiber by 19.1%, 49.3%, and 46.8%, respectively.

Current studies on aramid fibers mainly focus on the macroscopic processes of thermal aging and physical and chemical modification; however, the micromechanistic effect on the mechanical properties of these fibers is rarely reported, despite moisture being an important factor affecting the performance of transformer oil-paper insulating systems. Most studies of the aramid fiber have been conducted from the macroscopic point of view, but the mechanism of the effect of moisture on aramid fiber properties cannot be analyzed from the macroscopic angle.

Using molecular dynamics (MD), a cost-effective scientific tool, the property changes of a substance and their microscopic mechanisms [24,25,26,27,28,29] can be studied at the molecular and atomic levels. In this work, the effect of moisture on the properties of meta-aramid fiber insulating paper was studied using MD to explore the micromechanism. Seven models of meta-aramid fibers with different moisture contents were constructed, and dynamic simulation of each was carried out using *Materials Studio* software (Accelrys, San Diego, CA, USA). Variations in mechanical properties and thermal stabilities of the mixed models, performance parameters of the hydrogen bonding, as well as the related mechanisms were analyzed.

## 2. Model Calculation and Parameter Analysis

### 2.1. Modeling

In long-term operation of an oil-immersed transformer, the moisture content of the insulating paper may increase to 5% [17,30], or even higher in extreme cases. Considering that meta-aramid fibers have excellent structural stability and high temperature resistance, appropriate moisture contents were selected for comparison. Meta-aramid-H_2_O composite models with moisture contents of 0%, 1%, 2%, 3%, 4%, 5% and 9% were constructed; to facilitate description, these models are respectively marked as P_W_0_, P_W_1_, P_W_2_, P_W_3_, P_W_4_, P_W_5_, and P_W_9_.

The Visualizer module in the *Materials Studio* software package was first used to construct meta-aramid fibers with a degree of polymerization of 20. The water molecule model was then constructed, and, finally, the composite models were built using the Amorphous Cell module. To eliminate boundary effects and maintain a constant system density, a periodic boundary condition was adopted, with the density set at 1.4 g/cm^3^ [31]. 

**Figure 1:**
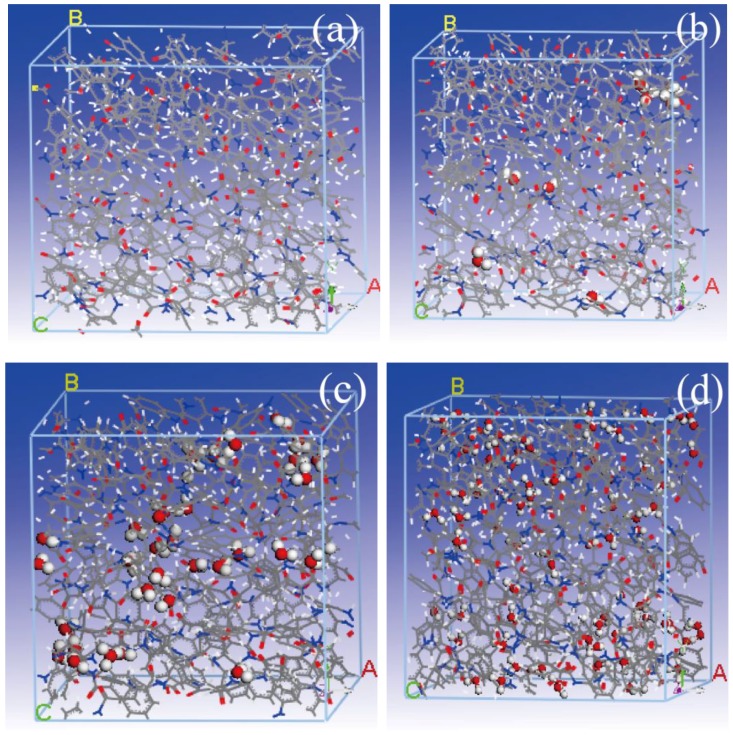
(**a**) P_W_0_, (**b**) P_W_1_, (**c**) P_W_4_ and (**d**) P_W_9_ models of meta-aramid fiber molecules.

Considering the situation in which the motion of water molecules is random when the moisture content is low, the water-free model (P_W_0_) and the composite model with a moisture content of 4% (P_W_4_) were adopted as the study and comparison models. Considering the temperature environment of a transformer oil-paper insulation system, the simulated temperatures were set at 343, 363, 383, 403 and 423 K for the study of the effect of temperature on the mechanical properties of the meta-aramid fibers. The P_W_0_, P_W_1_, P_W_4_ and P_W_9_ models are shown in Figure 1a–d respectively.

### 2.2. Molecular Dynamics Analog Calculation

Energy minimization (Minimizer) of the constructed composite models was carried out using the Discover module to ensure that the energy of each constructed system was in the lowest state. Smart Minimizer was adopted for the energy minimization, the convergence level was selected as Medium, and the maximum number of iterations was set at 5000. When a more stable model was obtained, annealing treatment of the system was carried out using the Forcite module. Annealing was carried out four consecutive times at a temperature in the range of 300 to 900 K. The systems were then considered to be at thermodynamic equilibrium.

The MD simulations were carried out after attaining thermodynamic equilibrium of the system. A 100 ps dynamic simulation was first carried out using the NVT (a certain particle number *N*, volume *V*, and temperature *T*) ensemble, and then a 100 ps dynamic simulation was performed using the NPT (a certain particle number *N*, pressure *P*, and temperature *T*) ensemble. The time step of the simulations was 1 fs. 

Both organic and inorganic components were involved in the model systems, so the condensed-phase optimized molecular potentials for atomistic simulation studies (COMPASS) force field [32], which is suitable for handling such systems, was selected. The Andersen thermostat and Berendsen barostat were adopted in the MD simulation. The Maxwell distribution was adopted for the distribution of the initial velocity of a molecule, and the velocity Verlet leapfrog integral method was adopted for the solution of Newton equations. The atom-based method was used for determining the Van der Waals and Coulomb forces, and the cut-off radius was set at 0.95 nm [33].

### 2.3. Rationality of Parameter Settings

**Figure 2:**
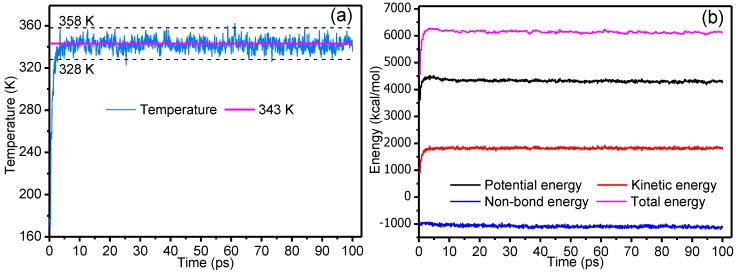
Basis for judgment of system stability. Variations of (**a**) temperature and (**b**) energies of mixed models with time.

Selection of the integration step and simulation time plays a critical role in the overall simulation experiment: an excessively long integration step will cause intense collisions between molecules, which makes the system data overflow, whereas the ability to search phase space is reduced by an excessively short integration step. In addition, if the simulation time is too short, the simulation may end before reaching thermodynamic equilibrium, whereas excessively long simulation times will inevitably lead to a waste of time. Two criteria were used for judging when a system had reached equilibrium: first, the temperature reached a balanced state (i.e., the standard deviation of temperature was within 15 K); second, the energy achieved a balanced state (i.e., the energy fluctuated [34] above and below a certain fixed value). Taking the P_W_4_ composite model in a 343 K simulated environment as an example, Figure 2a,b indicate the temperature-time and energy-time variations, respectively.

Figure 2 shows that the temperature of the whole system quickly reached a stable equilibrium state: potential energy, kinetic energy, and non-bonding energy quickly reached a balanced state without violent oscillations, which indicated that the selection of parameters of time step, total time of simulation, and other parameters were reasonable.

## 3. Results and Discussion

**Figure 3:**
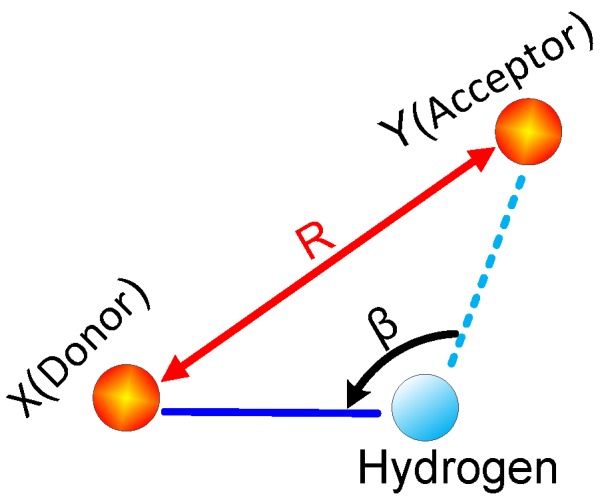
The definition of hydrogen bond. X represents the donor, which can form a chemical bond with a hydrogen atom; Y represents the acceptor, which can form a hydrogen bond with a hydrogen atom.

It is known from elastic mechanics that all mechanical parameters of a solid material can be theoretically calculated from the generalized Hooke’s Law matrix, and the mechanical properties of an object can be mainly described by the volume modulus (*K*), shear modulus (*G*), tensile modulus (*E*), and Cauchy pressure (*C*_12_*–C*_44_). There are two ways to define a hydrogen bond: the energy criterion and the geometric criterion. Geometric criteria have a relatively high degree of recognition, so geometric criteria were selected to calculate hydrogen bonds in this paper. The geometric criteria of hydrogen bond (X–H···Y) are shown in Figure 3. In this paper, the distance parameter *R* was set to 3 Å, and the angle β was set to 120°. Hydrogen bonding is a weak interaction force, intermediate between that of a chemical bond and a non-bond. Hydrogen bonding is also a special chemical bond [35,36,37,38,39] formed between atoms that have large electronegativities (such as O, F and S) combined with a hydrogen atom and a covalent bond to adjacent atoms with large electronegativity. Hydrogen bonds comprise both inter- and intramolecular interactions [33].

### 3.1. Effect of Temperature

**Figure 4:**
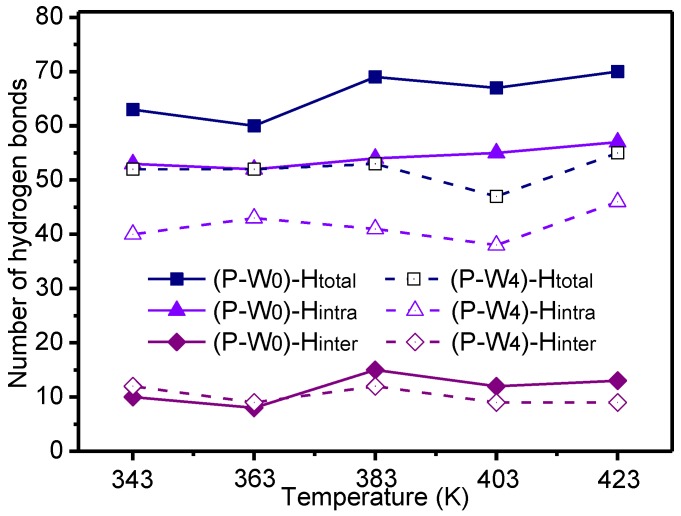
Number of hydrogen bonds at different temperatures for meta-aramid fiber molecules.

**Figure 5:**
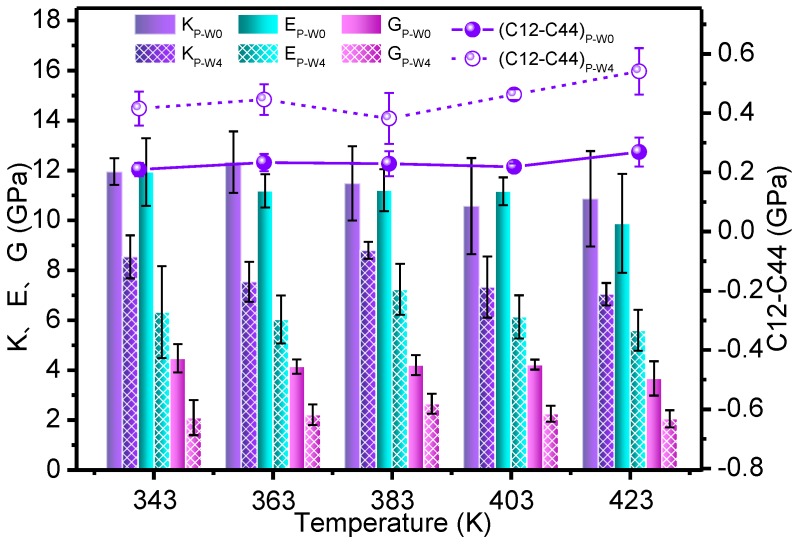
Mechanical parameters of meta-aramid fibers at different temperatures.

The number of hydrogen bonds and the mechanical parameters of the P_W_0_ and P_W_4_ models at different temperatures are indicated in Figure 4 and Figure 5, respectively.

Figure 4 shows that, with the temperature rise, there was no regularity in the number of inter- and intramolecular hydrogen bonds of the meta-aramid fibers. The temperature variation fluctuated slightly in each paired set of data. When the moisture content was 4%, the number of hydrogen bonds between the molecular chains of meta-aramid fibers was significantly lower than that in the anhydrous state; the difference between the average numbers of hydrogen bonds in the two conditions was 24.87%. Therefore, in a transformer environment, the impact of temperature on the hydrogen-bond network of meta-aramid fibers is not obvious, and moisture is an important factor damaging the hydrogen-bond network of the fibers.

The overall variations of Figure 5 showed that, with the increase of temperature, the parameters *K*, *E* and *G* decrease slightly, whereas *C*_12_*–C*_44_ showed a rising trend, though the change was relatively slow. In the temperature environment of a transformer, the mechanical properties of the meta-aramid fibers decrease slightly with a temperature rise. This is due to the arrangement of the molecular structure of the meta-aramid fibers and the existence of a large number of hydrogen bonds between the molecular chains. This result is consistent with the conclusions in the literature [40]. Compared with the P_W_0_ model, the tensile, volume, and shear moduli of the P_W_4_ model were small and the Cauchy pressure was high. These results show that the mechanical properties of the meta-aramid fibers were lowered by the presence of water. A combined analysis of the data in Figure 4 shows that the main reason for this observation is that the intermolecular hydrogen-bond network of the meta-aramid fibers was destroyed, and the interactions between the fiber chains were reduced by the presence of moisture.

The above data showed that, in the operating environment of a transformer, a rise in temperature will lead to a decrease of the mechanical properties of the meta-aramid fibers; specifically, the intermolecular hydrogen-bond network of the meta-aramid fibers is destroyed due to the presence of moisture, which affects these properties.

As one of the main aging products of a transformer oil–paper insulation system, moisture is an important factor affecting the mechanical properties of the aramid fibers. Considering the common hot-spot temperature of power transformers, the temperature was set at 403 K for further dynamic simulations. The mechanism of the effect of moisture on the mechanical properties and thermal stability of the meta-aramid fibers was further explored in this condition.

### 3.2. Analysis of Hydrogen Bonds

**Figure 6:**
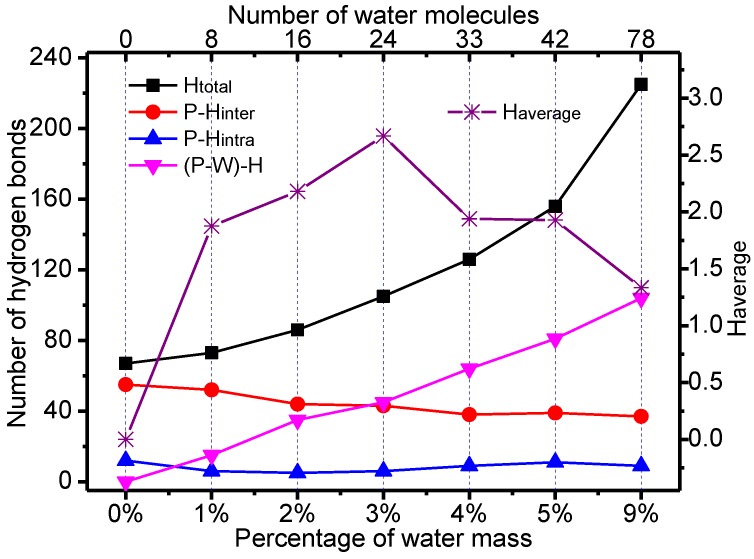
Hydrogen bond characteristics of composite models with different moisture contents.

Figure 6 shows the statistical characteristics of the hydrogen bonds of the meta-aramid fibers with different moisture contents.

It can be seen that water molecules and meta-aramid fibers form a large number of hydrogen bonds; the number of hydrogen bonds ((P–W)–H) formed with water molecules and meta-aramid fibers showed a rising trend with increasing water content. The number of intermolecular hydrogen bonds (P–H_inter_) in the meta-aramid fibers was reduced, and the number of intramolecular hydrogen bonds (P–H_intra_) of the fibers remained unaffected. Compared with the P_W_0_ model, the moisture contents of the composite models were 1%, 2%, 3%, 4%, 5% and 9%, and their respective percentage decreases in numbers of intermolecular hydrogen bonds were 10.34%, 21.14%, 25.86%, 34.45%, 32.76% and 36.21%. In general, the number of hydrogen bonds between meta-aramid fiber chains decreased with the increase in moisture content. 

There is an important relationship [33] between the hydrogen bonds formed between the high molecular mass polymer and their mechanical properties. It can be seen that the positions of the fibers that are first affected by moisture are those located between the molecular chains. With the increase of moisture content, the hydrogen bond network of meta-aramid fiber was destroyed, and its mechanical properties reduced. Similar conclusions can be found in the literature [30]. Through the analysis of the average number of hydrogen bonds (H_average_) formed between the water molecules and meta-aramid fibers, a downward trend was observed after the upward trend. The H_average_ was at a maximum when the moisture content was 3%, indicating that the hydrogen bonding between water molecules and meta-aramid fibers reached saturation. When the moisture content was less than 3%, the binding effect of the meta-aramid fibers on moisture increased with increasing moisture content; when hydrogen bonding between moisture and meta-aramid fibers reached saturation, the binding effect of the fibers on the water molecules started to decrease. This can explain why, when the moisture content exceeds 3%, the fiber structures are damaged by moisture, the damage is more severe with increasing moisture content, and the combined stability of the meta-aramid fiber chains and water molecules will gradually decrease.

### 3.3. Free Volume

In accordance with the free volume theorem, the volume of high molecular mass polymers comprises two parts: the occupied volume (*V_O_*) of the macromolecules and the so-called empty volume not occupied by the macromolecules. Fractional free volume (*FFV*) is the ratio [41] of the free volume (*V_F_*) to the total volume of these polymers. The free volume of a high molecular mass polymer has an important effect on the diffusion of small molecules. In this work, the Van der Waals radius of H_2_O in the meta-aramid fiber system was calculated using the hard-ball probe model [42], and the free volume of the water molecules was calculated from the Van der Waals radius. The calculated results are shown in Table 1.

**Figure 7:**
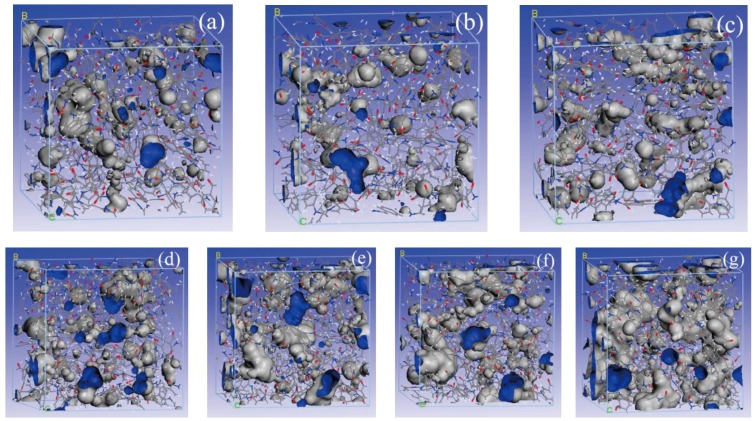
Free volumes of composite models with different moisture contents. (**a**) P_W_0_; (**b**) P_W_1_; (**c**) P_W_2_; (**d**) P_W_3_; (**e**) P_W_4_; (**f**) P_W_5_; (**g**) P_W_9_.

Figure 7 shows a schematic diagram of the free volume of meta-aramid fibers with different moisture contents.

From the data in Table 1, the *FFV* values of meta-aramid fibers with different moisture contents were 1% < 0% < 2% < 3% < 4% < 5% < 9%. From the fractional relationship of free volume, it can be concluded that *FFV* generally rose (i.e., the free volume fraction of meta-aramid fibers increased) with the increase of moisture content. What is noteworthy is that *FFV* value of the P_W_1_ composite model was lower than that of P_W_0_. The main reason is that the intermolecular interaction of the meta-aramid fibers was strengthened for hydrogen bonds formed between water molecules and meta-aramid fibers in the case of the low-water (1%) condition, and, simultaneously, the molecular chain structure of the meta-aramid fibers became more compact under the action of molecular thermal motion. With an increase of moisture content, the empty-volume transition of water molecules between the meta-aramid fiber molecules was more active, hydrogen bonding formed between the water molecules and the O and N atoms on the meta-aramid fiber chain, and the interaction between the original fiber chains weakened. Once a large number of hydrogen bonds formed between water molecules and fibers, the hydrogen-bond network of the molecular chains of the fibers became damaged, and their free volume increased.

### 3.4. Thermal Stability of Meta-Aramid Fibers and Water Molecule Diffusion

A statistical analysis was carried out to track the movements of the system, and the thermal stability and kinetic characteristic parameters were obtained. The microscopic conditions of the cellulose of the insulating paper under thermal motion can be inferred from the motion of the molecular chains. The mean square displacement (MSD), which reflects the center mass displacement of molecular chains in a single time period, is an important parameter used to describe chain movement. The expression for MSD is given as:

(1) $$\mathrm{MSD}=\langle |{\vec{r}}_{i}(t)-{\vec{r}}_{i}(0){|}^{2}\rangle$$

where ${\vec{r}}_{i}(t)$ represents the position of atom *i* at time *T* in the system, and ${\vec{r}}_{i}(0)$ represents the initial position of the atom.

**Figure 8:**
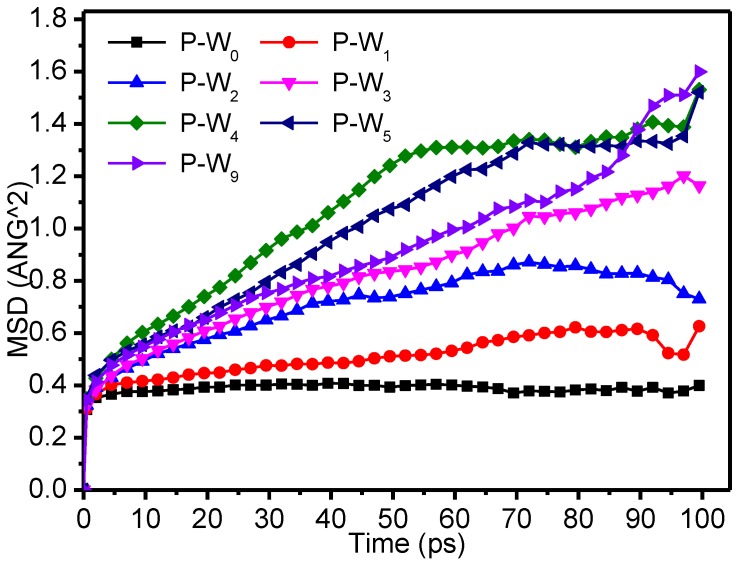
Mean square displacement (MSD) of the molecular chains of meta-aramid fibers with different moisture contents.

Figure 8 shows the variation of the MSD of the molecular chains of meta-aramid fibers with time for different moisture contents. In general, the MSD increased with increasing moisture content: for low moisture content, the MSD value was relatively small, whereas when the moisture content exceeded 5%, the movement of the fiber chains was relatively violent.

The radius of gyration was further utilized to confirm that the movement of the fiber chains was affected by moisture. The average value for the radii of gyration of three chains was taken. The diffusion coefficient can directly reflect violent movement of water molecules, that is, the greater their diffusion ability, the weaker the binding ability of meta-aramid to the water molecules. Einstein’s relation was used to describe the movement of water molecules in the aramid fiber. The radii of gyration (1/Å) for different moisture contents and the calculated values of the diffusion coefficients *D*_w_ (10^−12^ m^2^·s^−1^) are listed in Table 2.

**Figure 9:**
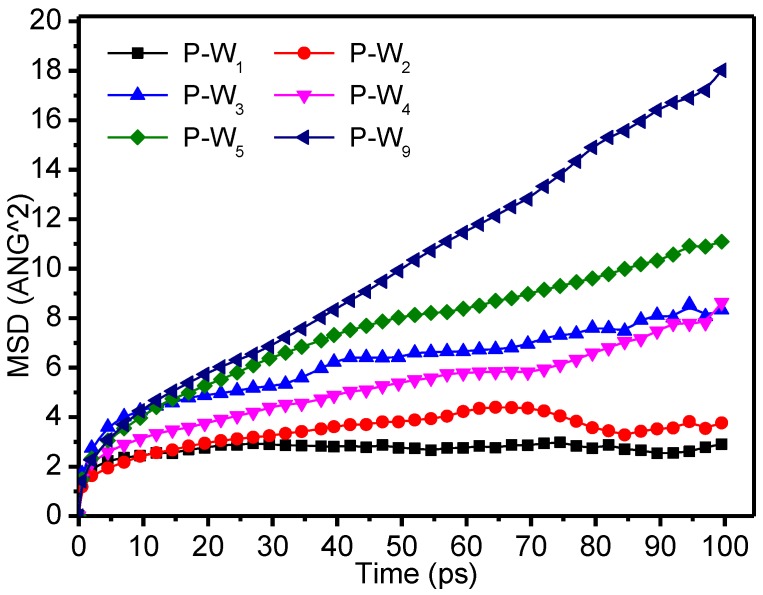
Mean square displacement of different moisture contents.

**Figure 10:**
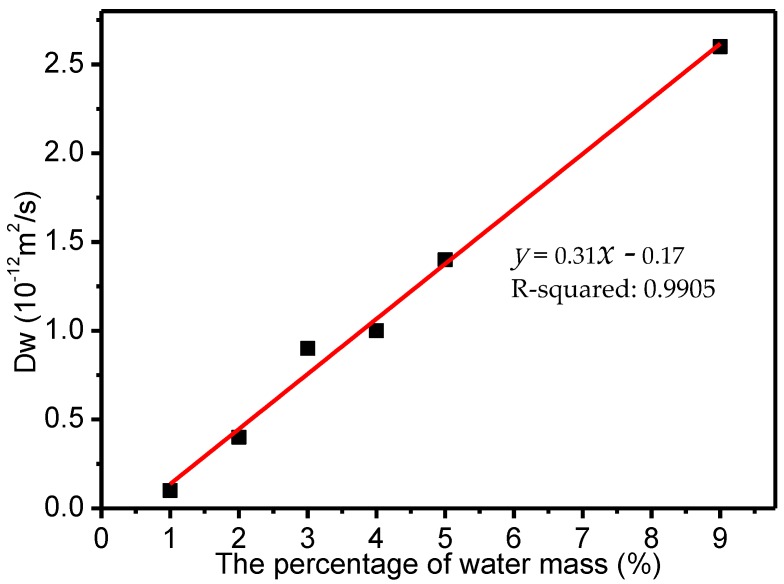
Linear fitting of water molecule diffusion coefficients as a function of water content.

Table 2 shows that the gyration radii generally increased with the increase of moisture in the fibers. Figure 9 shows that the MSD of the water molecules increased with increasing moisture contents with time. Linear fitting, as shown in Figure 10, was carried out to determine the relationship between the water diffusion coefficients and the water contents, based on the data in Table 2. The linear relationship was defined by *y* = 0.31*x* − 0.17, that is, the weakening of the water molecule binding capacity of the meta-aramid fibers was described by a linearly decreasing relationship. The simulated R-squared was 0.9905, confirming the credibility of the fitted results.

When moisture increased, the fractional free volume of the fibers increased, and the interaction between the fiber chains decreased, which was reflected in the increase of the gyration radius (i.e., the chain motion increased). At the same time, the interaction between moisture and meta-aramid fibers decreased with increasing moisture, the free movement of the water molecules increased, and the relationship between the diffusion coefficients of the water molecules and the moisture content was represented by a linear increase with a slope of 0.31. Water molecules moved into the voids between the meta-aramid fibers, thereby accelerating damage to the original hydrogen-bond network of the fibers and reducing their thermal stability.

### 3.5. Analysis of Mechanical Properties

As for the insulating paper used in power transformer, the deterioration of its mechanical properties is the main factor that influences the insulating performance [6,20]. Table 3 shows the mechanical parameters of meta-aramid fibers under different moisture contents.

Analysis of Table 3 shows that the volume and tensile moduli of the meta-aramid fibers decreased with the increase in moisture content of the composite models, meaning that their rigidity weakened, their Cauchy pressure increased, and their ductility was enhanced. Comparing fiber with 2% moisture with fiber in an anhydrous state, the tensile and volume moduli decreased by 41.12% and 22.39%, respectively. In extreme cases where the moisture contents in the composite models were 5% and 9%, the tensile moduli decreased by 63.57% and 59.47%, respectively, and the bulk moduli decreased by 40.9% and 52.19%, respectively. These results indicate that the mechanical properties of the meta-aramid fibers diminished with increasing moisture content.

To further study this relationship, the correlation between the mechanical properties and intermolecular hydrogen bonds of meta-aramid fibers was analyzed using SPSS software (SPSS, Chicago, IL, USA). The Poisson correlation between the tensile modulus and intermolecular hydrogen bonds of meta-aramid fiber chains was 0.919, with a significance of 0.003; the Poisson correlation between bulk modulus and intermolecular hydrogen bonds of the fiber chains was 0.921, with a significance of 0.003. In both cases, the significance was far lower than 0.05, which showed that the relationship between the intermolecular hydrogen bonds of the fiber and its mechanical properties is significant, and, therefore, the destruction of the hydrogen-bond network between the molecular chains of the fiber will lead to degradation of its mechanical properties. 

This data analysis showed that, with an increase of moisture content, the rigidity of meta-aramid fibers was decreased and ductility was reinforced. In the research environment of this study, the effect of moisture on the mechanical properties of meta-aramid fibers was significant.

## 4. Conclusions

Using the molecular dynamics method, the effect of moisture on the thermal stability and mechanical properties of meta-aramid fibers, and the associated micromechanism were analyzed by considering the characteristics of the hydrogen-bond network, microscopic motion, and free volume. The following conclusions were obtained:

1. Through the analysis of the hydrogen-bond networks of the composite models, it was found that the hydrogen bonds between the molecular chains of the meta-aramid fibers were first affected by moisture. Damage to this network resulted in a decrease in mechanical properties. With increasing moisture content, a large number of hydrogen bonds formed between the water molecules and meta-aramid fibers, which damaged the hydrogen bonds in both the intra- and intermolecular chains of the fibers. Therefore, protection of the intermolecular hydrogen-bond network of meta-aramid fibers from water molecules is a feasible method for improving their mechanical properties under certain water contents.
2. The thermal stability of meta-aramid fibers was decreased by the presence of moisture. The free volume of the meta-aramid fibers increased with increasing moisture content, the interactions between the fiber chains weakened, and this led to an increase in chain motion; in contrast, the linear relationship between the water molecule diffusion coefficients and moisture content, characterized by a slope of 0.31, showed that the rate of damage to the original hydrogen-bond network of the fibers increased, their chain movement increased further, and their thermal stability decreased.
3. In general, the mechanical properties of meta-aramid fibers decreased with an increase in moisture content. Compared with the water-free model, the tensile and bulk moduli of the model with a moisture content of 9% decreased by 59.47% and 52.19%, respectively. Damage to the hydrogen-bond network is an important factor leading to reduction in mechanical properties.

## Figures and Tables

**Table 1:** Free volumes of meta-aramid fibers with different moisture contents (Å^3^).

Volume	P_W_0_	P_W_1_	P_W_2_	P_W_3_	P_W_4_	P_W_5_	P_W_9_
*V*_F_	848.56	675.14	1058.37	1086.89	1482.10	1517.35	2607.84
*V*_O_	16329.32	16364.57	16565.28	16740.05	16666.25	1671.07	16502.19
*FFV (%)*	4.49	3.86	6.00	6.09	8.17	8.29	13.64

**Table 2:** Gyration radii of meta-aramid fibers and diffusion coefficients of water molecules determined for the model compositions.

R and *D*_W_	P_W_0_	P_W_1_	P_W_2_	P_W_3_	P_W_4_	P_W_5_	P_W_9_
*R*	65.91	66.67	66.83	67.01	66.60	67.48	67.80
*D*_W_	-	0.1	0.4	0.9	1	1.4	2.6

**Table 3:** Mechanical properties (G·Pa) of meta-aramid fibers with different moisture contents.

Mechanical Parameters	P_W_0_	P_W_1_	P_W_2_	P_W_3_	P_W_4_	P_W_5_	P_W_9_
*K*	11.88	11.76	9.22	10.37	7.17	7.02	5.68
*E*	11.72	8.22	6.90	5.24	6.46	4.27	4.75
*C*_12_–*C*_44_	0.21	0.37	0.40	0.54	0.48	0.75	0.63
